# Topography-Guided Custom Ablation Treatment for Post-Traumatic Corneal Irregularities—Case Reports

**DOI:** 10.3390/biomedicines13081818

**Published:** 2025-07-24

**Authors:** Łukasz Drzyzga, Dorota Śpiewak, Mariola Dorecka, Dorota Wyględowska-Promieńska

**Affiliations:** 1 Department of Ophthalmology, Prof. K. Gibiński University Clinical Center, Medical University of Silesia, 40-514 Katowice, Poland; 2 Clinical Ophthalmology Centre OKOLUX, 40-514 Katowice, Poland; 3 Department of Ophthalmology, Faculty of Medical Sciences in Katowice, Medical University of Silesia, 40-514 Katowice, Poland

**Keywords:** topography-guided custom ablation treatment, regularization of the corneal surface, post-traumatic corneal irregularities

## Abstract

**Background:** Post-traumatic corneal wounds that require suturing are quite common; they reduce corneal transparency and cause corneal distortion, leading to corneal astigmatism and higher-order aberrations. Excimer laser treatment can be a potentially beneficial intervention for such wounds. The observation aimed to evaluate the effectiveness of topography-guided custom ablation treatment (TCAT) in patients with corneal injuries. **Methods:** This observation included three patients with corneal penetrating trauma (full-thickness corneal scar) and one patient with corneal blunt trauma, i.e., a non-penetrating injury with corneal laceration (partial-thickness corneal scar). This cohort study was conducted from July 2021 to August 2023. After first-stage treatment (stabilization of the post-traumatic visual defect confirmed by refraction and topography examination, corneal healing, and improvement of the corneal scar), the patients underwent the second-stage treatment, i.e., TCAT with a 20 to 45 s application of mitomycin C solution to avoid haze induction. After TCAT, the uncorrected distance visual acuity (UDVA) and best-corrected distance visual acuity (BCVA) were measured. Refractive astigmatism was assessed using autorefractometry. Topographic astigmatism was analyzed using corneal topography and pachymetry. The root mean square (RMS) of the higher-order aberration was calculated using Zernike coefficients. The patients’ corneal healing and refractive changes were monitored. **Results:** All patients were monitored for corneal healing and refractive changes and underwent the same second-stage treatment, which utilized TCAT to regularize the corneal surface and reduce higher-order aberrations (HOAs). The UDVA of patients 1, 2, 3 and 4 improved by 3, 7.5, 4 and 6 rows (Snellen chart), respectively. The resultant UDVA was 1.0, 0.9, 0.7 and 1.2, while BCVA was 1.0, 1.2, 1.0, and 1.5, respectively. **Conclusions:** TCAT regularized the patients’ corneal surfaces and reduced HOAs. We, therefore, conclude that TCAT may be a beneficial second-stage treatment for corneal trauma-induced astigmatism.

## 1. Introduction

Severe corneal injuries, such as penetrating wounds, can lead to vision loss or eye loss. Full-thickness corneal wounds typically require suturing, except for wounds that are water-tight and do not disrupt the corneal curvature. More extensive corneal wounds require optimal reconstruction, especially if accompanied by iris prolapse, as inadequate closure has permanent adverse effects that impair vision. The correct corneal wound suturing technique accelerates wound healing, corneal transparency recovery, and ensures that the corneal curvature is as close to the natural shape as possible. Additionally, the correct suturing technique for full-thickness wounds prevents iris–corneal adhesions. Correct primary suturing of penetrating corneal wounds significantly influences the treatment outcome, particularly visual acuity, and provides an opportunity for a subsequent laser procedure involving corneal surface regularization, which may even restore full visual acuity [1].

The cornea is the main refractive surface of the eye and accounts for a significant proportion of congenital and acquired ocular aberrations. Correcting higher-order aberrations (HOAs) involves aberrometry applications, such as wavefront-guided (WFG) and wavefront-optimized (WFO) ablation profiles, as well as topography-guided custom ablation treatment (TCAT). WFO profile treatment aims to reduce spherical aberrations without altering the existing HOAs, while TG interventions correct spherical aberrations, HOAs, and pre-existing corneal surface irregularities [2].

TCAT modifies the cornea by treating anatomical changes. Topography-guided (TG) systems generate ablation profiles by using corneal topography data. The profiles are modified to improve corneal irregularities, correct corneal shape abnormalities, and optimize corneal curvature. The topography measurements are reproducible, and the corneal curvature assessment is not dependent on pupil size, rendering TG ablation independent of pupil centre shift errors. The preoperative evaluation involves measuring additional points on the cornea, which enables treatment of the peripheral cornea, responsible for most HOAs. Another advantage of TG treatment is its use in eyes with high aberration and corneal opacities, where an aberrometer may yield inaccurate measurements. This renders TG data more reproducible and reliable than wavefront-derived data. Therefore, TCAT is an ideal platform for improving corneal symmetry, correcting lower- and higher-order aberrations, and enhancing visual acuity and quality [2,3], while avoiding high-risk corneal transplantation in certain patients.

The primary objective of this study was to assess the efficacy of topography-guided custom ablation treatment in enhancing visual acuity outcomes, specifically uncorrected distance visual acuity (UDVA) and best-corrected visual acuity (BCVA), in patients with post-traumatic corneal irregularities at 6 months post-treatment. Secondary objectives included evaluating the treatment’s impact on other clinical parameters, namely spherical refraction, cylindrical refraction, topographic astigmatism, mean keratometry, corneal thickness, and root mean square (RMS) HOAs. A secondary objective was to investigate changes in Halo/Glare Status, stratified by corneal injury type, to determine the effect of the treatment on visual disturbances.

## 2. Materials and Methods

The study analyzed data from four patients. One patient received only pharmacological treatment in the first stage, as the injury was non-penetrating and did not require surgical intervention. Three patients with full-thickness corneal wounds underwent emergency surgery within the first 24 h after injury. The wounds were sutured with single Ethilon 10/0 sutures by the same surgeon. After corneal wound repair, the patients received the ophthalmic levofloxacin solution (5 mg/mL, Oftaquix^®^; Santen Oy, Tampere, Finland) into the conjunctival sac four times a day for the first week, followed by steroid anti-inflammatory eye drops (Dexamethasone^®^ 0.1%; Polfa S.A., Warsow, Poland) four times a day for the first month, followed by a reduction of 1 drop every month. Lubricating eyedrops (Systane Hydration^®^; Alcon Laboratories, Inc., Fort Worth, TX, USA) were administered five times daily. Clinical parameters were measured at least 6 months after injury, when visual impairment and corneal scarring had stabilized and TCAT could be utilized.

The uncorrected distance visual acuity (UDVA) and best-corrected distance visual acuity (BCVA) were measured. Refractive astigmatism was assessed by autorefractometry using the Dioptron Auto Ref-Keratometer Huvitz HRK-8000A. Topographic astigmatism was analyzed with the Oculus Pentacam, a non-invasive device that determines the topography and pachymetry of the entire cornea using a 360° rotating Scheimpflug camera. Refractive maps, keratometry, and pachymetry were obtained. The RMS HOA was calculated using Zernike coefficients.

TCAT was performed as the second-stage treatment, not earlier than 6 months after the first-stage treatment, using the Schwind Amaris excimer laser. Local anaesthesia (0.5% proxymetacaine hydrochloride, Alcaine^®^; Alcon Laboratories, Inc., Fort Worth, TX, USA) was administered, followed by TCAT (Schwind Amaris 500e excimer laser software), taking into account the mean corneal epithelium thickness based on the corneal epithelial map. The optic zone diameter was 6.7–7.0 mm, and the transition zone was 1.04–1.26 mm. The current lasers use an eye-tracking system that monitors eye movements in real-time (including saccadic, vestibular, optokinetic, vergence, and cyclotorsion movements) and switches off the light beam when the software deems it necessary. Additionally, these lasers use a so-called closed-loop tracking system, which locks the device on the eye, and an open-loop tracking system. This video system attempts to follow rather than compensate for eye movements and detects the pupil position in the infrared spectrum.

**Figure 1:**
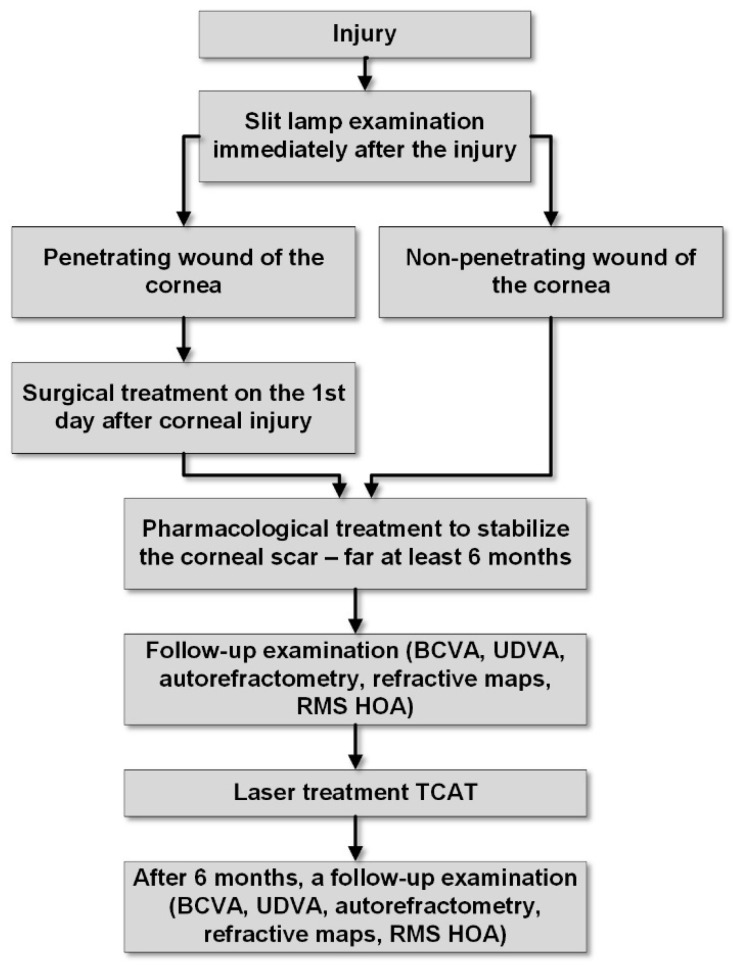
Diagram of therapeutic procedures.

Mitomycin C solution (0.02%) was applied immediately after surgery for 20–45 s to avoid haze induction. The topical broad-spectrum antibiotic levofloxacin solution (5 mg/mL, Oftaquix^®^; Santen Oy, Tampere, Finland) was prescribed to be applied to the conjunctival sac four times a day for the first week, followed by steroidal anti-inflammatory eye drops (Dexamethasone^®^ 0.1%; Polfa S.A. Warsaw, Poland) four times a day for the first month, and lubricating drops (Systane Hydration^®^; Laboratories, Inc., Fort Worth, TX, USA) five times a day for one month and then as needed. Another examination with clinical parameter measurements was performed 6 months after the laser treatment, when the corneal healing process was complete. Figure 1 illustrates the successive stages of treatment for the presented patients in a diagram.

## 3. Results

### 3.1. Statistical Analysis

A significance level of α = 0.05 was adopted for all statistical inferences, corresponding to a 95% confidence level. Descriptive statistics for continuous variables were reported as means with standard deviations (SD) and ranges (minimum to maximum), while categorical variables were summarized as frequencies with percentages. For each continuous parameter, treatment effects were calculated as the mean difference $\bar{d}$ according to Formula (1):

(1) $$\bar{d}={\bar{x}}_{post}-{\bar{x}}_{pre}$$

where ${\bar{x}}_{post}$ and ${\bar{x}}_{pre}$ are the means of the post-treatment and pre-treatment observations, respectively, computed according to Formula (2):

(2) $$\bar{x}=\frac{\sum_{i=1}^{n}x_{i}}{n}$$

with $x_{i}$ representing individual observations and n the sample size.

However, for parameters measured in diopters, the change in (1) is calculated as the absolute difference, given by

$$\bar{d}=|{\bar{x}}_{post}-{\bar{x}}_{pre}|$$

The SD of the differences (${SD}_{diff}$) was calculated by Formula (3):

(3) $${SD}_{diff}=\sqrt{\frac{\sum_{i=1}^{n}{\left(d_{i}-\bar{d}\right)}^{2}}{n-1}},d_{i}=x_{i,post}-x_{i,pre}$$

The standard error of the mean difference ${SE}_{diff}$ was derived from Formula (4):

(4) $${SE}_{diff}=\frac{{SD}_{diff}}{\sqrt{n}}$$

The 95% CI for the mean difference was calculated using the t-distribution critical value for a 95% confidence level $t_{0.975,\;df=n-1}$ with *n* − 1 degrees of freedom using Formulas (5a) and (5b):

(5a) $${CI}_{lower,diff}=\bar{d}-t_{0.975,df=n-1}\times {SE}_{diff}$$

(5b) $${CI}_{upper,diff}=\bar{d}+t_{0.975,df=n-1}\times {SE}_{diff}$$

Finally, the effect size was quantified using Cohen’s d according to Formulas (6a) and (6b):

(6a) $$\text{Cohen’s}\;d=\frac{\bar{d}}{{SD}_{pooled}}$$

(6b) $${SD}_{pooled}=\sqrt{\frac{{SD}_{pre}^{2}+{SD}_{post}^{2}}{2}}$$

where ${SD}_{pre}$ and ${SD}_{post}$ are the SDs of the pre-treatment and post-treatment observations, respectively. Cohen’s *d* was interpreted as small (0.2), medium (0.5), or large (0.8). Statistical significance was determined by whether the 95% CI for the mean difference excluded zero.

The analysis was conducted according to the RECORD (Reporting of Studies Conducted Using Observational Routinely Collected Health Data) guidelines [4] (Benchimol et al., 2015) and the CARE (Case Reports) guidelines [5] (Gagnier et al., 2013).

Analyses were conducted in R Statistical Software (version 4.3.3; R Core Team, 2024) on Windows 11 Pro 64 bit (build 26100) [6], using the following packages: ggalluvial (version 0.12.5; Brunson JC, Read QD, 2023) [7], report (version 0.5.8; Makowski D et al., 2023) [8], gtsummary (version 2.2.0; Sjoberg D et al., 2021) [9], ggplot2 (version 3.5.0; Wickham H, 2016) [10], dplyr (version 1.1.4; Wickham H et al., 2023) [11], and tidyr (version 1.3.1; Wickham H et al., 2024) [12].

### 3.2. Characteristics of Socio-Demographic and Clinical Parameters at Baseline (preTCAT Treatment) Time Point

Table 1 provides baseline characteristics of four male patients with post-traumatic corneal irregularities, with a mean age of 44 years (SD = 3.56), and an age range of 41 to 48 years. Injury characteristics included penetrating wounds of the cornea in three patients (75.0%) and non-penetrating corneal trauma in one (25.0%). All patients underwent laser treatment using TCAT, with three having prior emergency surgical suturing (75.0%) and one receiving no emergency surgical intervention (25.0%). Baseline clinical parameters showed a mean UDVA of 0.44 (SD = 0.26, range 0.15 to 0.70) and BCVA of 0.83 (SD = 0.24, range 0.50 to 1.00) in decimal notation, reflecting moderate to poor visual acuity. Spherical refraction averaged 0.88 D (SD = 1.64, range −1.50 to 2.25), cylindrical refraction −2.69 D (SD = 0.72, range −3.75 to −2.25), and topographic astigmatism 2.63 D (SD = 0.50, range 1.90 to 3.00), indicating significant astigmatism. The mean keratometry was 42.86 D (SD = 1.81, range 41.00 to 45.00), the mean corneal thickness was 522.75 µm (SD = 53.04, range 446.00 to 568.00), and the mean RMS higher-order aberration was 1.25 µm (SD = 1.22, range 0.25 to 3.02). Halo/Glare Status was positive in three patients (75.0%) and negative with decreased visual acuity in one (25.0%).

### 3.3. Graphical and Tabular Presentation of Treatment Results

Table 2 presents the pre-TCAT results, refractive maps, and Zernike analyses based on the Pentacam scans, and Table 3 presents the post-TCAT results, refractive maps, and Zernike analyses based on the Pentacam scans.

Patients 1, 2, 3, and 4 had the UDVA of 1.0, 0.9, 0.7, and 1.2 (Snellen chart), respectively, while the respective BCVAs were 1.0, 1.2, 1.0, and 1.5.

**Figure 2:**
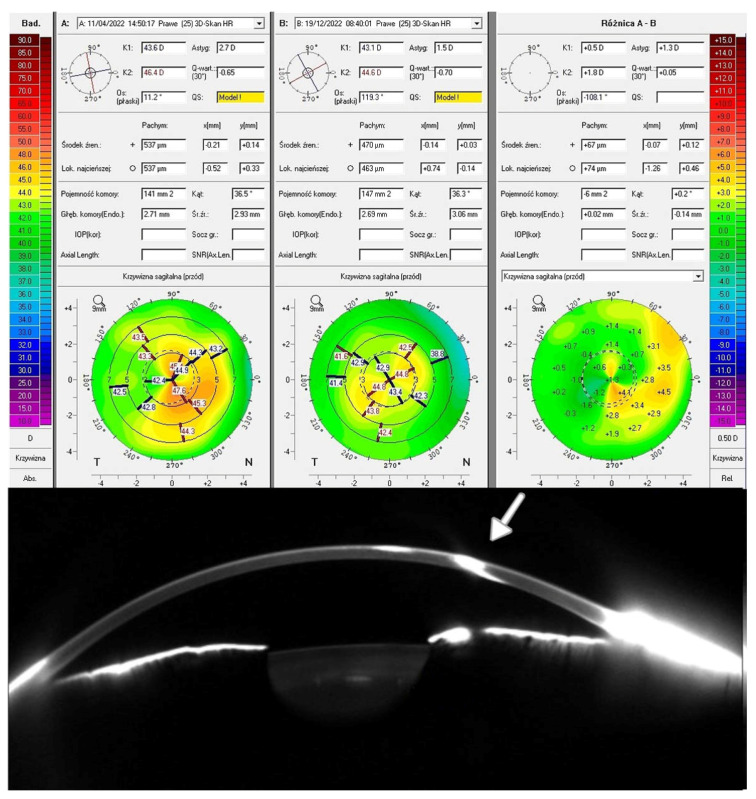
Patient 3—the top of the figure shows the scans of the Pentacam test: top-left—before treatment; top-middle—after treatment; top-right—the difference in refraction results before and after treatment. The bottom part shows the corneal tomography image. The white arrow indicates the site of injury.

Figure 2 displays the Pentacam cornea maps of one of the studied patients (Patient 3), based on elevation data derived from Scheimpflug images obtained before and after TCAT. Visual acuity improved due to corneal surface regularization and the associated reduction in topographic astigmatism and RMS HOAs. The remaining results for the treated patients are presented in the Supplementary Material Figures S1–S3.

All patients experienced improved visual acuity following surgical and laser treatment. The UDVA and BCVA improved by 0.7–1.2 and 1.0–1.5 (Snellen chart), respectively. Furthermore, topographic and refractive astigmatism was reduced. Three eyes had a significantly reduced RMS HOA. Laser treatment of post-traumatic corneal lesions improves visual acuity and quality due to the reduction in higher-order aberrations, especially coma.

### 3.4. Six-Month Outcomes of Topography-Guided Custom Ablation for Post-Traumatic Corneal Irregularities

Table 4 reports changes in clinical parameters 6 months after topography-guided custom ablation treatment.

#### 3.4.1. UDVA Changes

**Figure 3:**
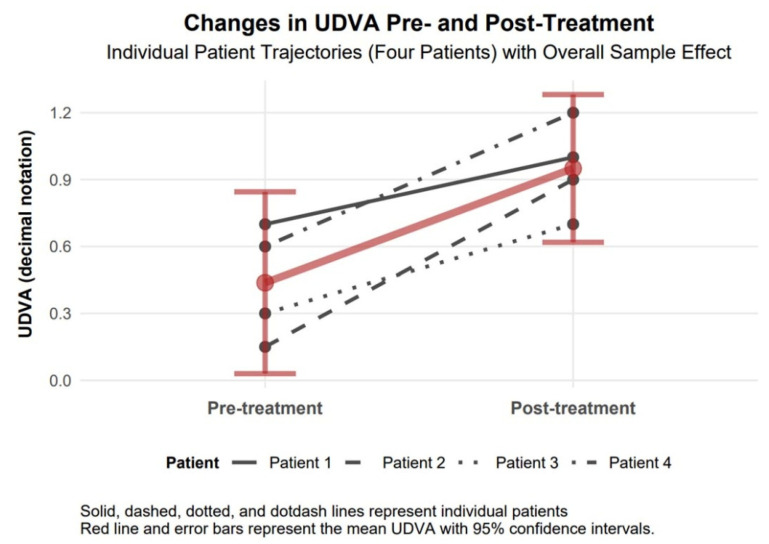
Changes in UDVA 6 months after topography-guided custom ablation treatment compared to pre-treatment (N = 4).

UDVA (decimal notation) increased by 0.51 (95% CI of 0.19 to 0.83; Cohen’s d = 2.20), a statistically significant improvement as the CI excludes zero, with a large effect size. In decimal notation, UDVA represents visual acuity, where higher values indicate better vision (e.g., 1.0 is 20/20, 0.5 is 20/40). The mean baseline UDVA of 0.44 (equivalent to approx. 20/45) improved to 0.95 (approx. 20/21), reflecting a substantial gain in uncorrected visual acuity, which enhances patients’ ability to perform daily tasks without correction (Figure 3).

#### 3.4.2. BCVA Changes

**Figure 4:**
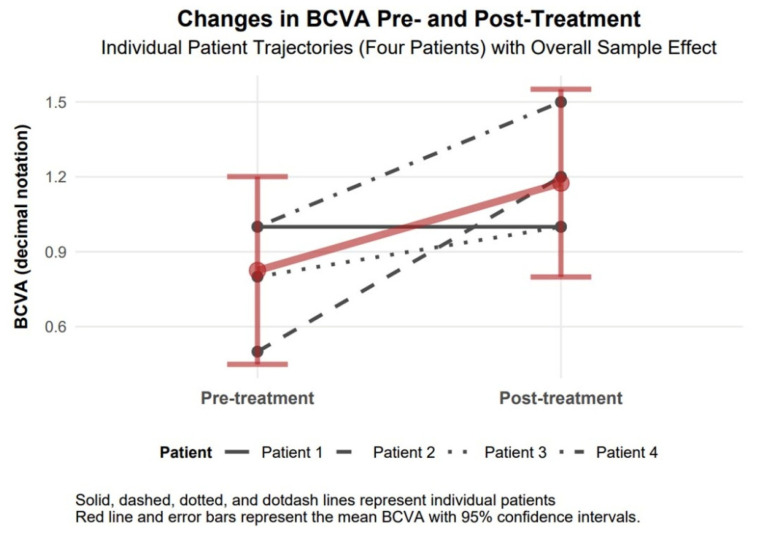
Changes in BCVA 6 months after topography-guided custom ablation treatment compared to pre-treatment (N = 4).

The baseline mean BCVA of 0.83 (approximately 20/24) improved to 1.18 (approximately 20/17) following treatment, indicating a notable enhancement in corrected visual acuity among the four patients. This change, while not statistically significant due to a wide confidence interval, represents a clinically relevant improvement as it approaches or exceeds normal visual acuity (1.0). The large effect size (Cohen’s d = 1.48) further underscores the magnitude of this improvement, indicating a substantial treatment effect on best-corrected vision despite the lack of statistical reliability (Figure 4).

#### 3.4.3. Spherical Refraction Changes

**Figure 5:**
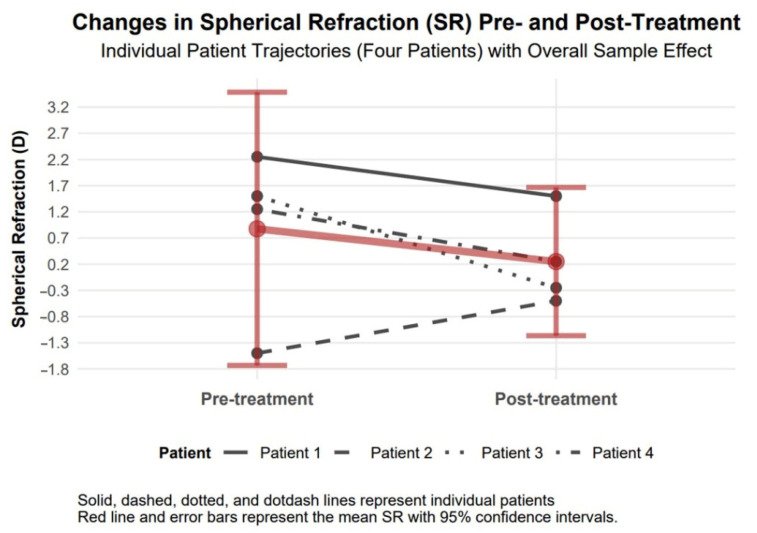
Changes in spherical refraction 6 months after topography-guided custom ablation treatment compared to pre-treatment (N = 4).

Spherical refraction exhibited an improvement of 1.00 diopters in absolute value (95% confidence interval: 0.68 to 1.32, Cohen’s d = 1.32), which confirms visual acuity improvement in our study patients (Figure 5).

#### 3.4.4. Cylindrical Refraction Changes

**Figure 6:**
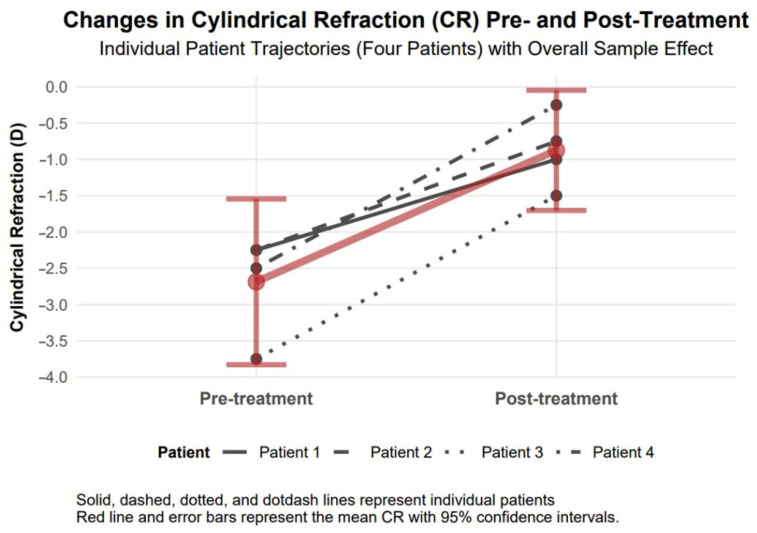
Changes in cylindrical refraction 6 months after topography-guided custom ablation treatment compared to pre-treatment (N = 4).

Cylindrical refraction showed a significant improvement of 1.81 diopters in absolute value (95% confidence interval: 0.99 to 2.63, Cohen’s d = 2.89), indicating a large effect size, with a decrease from −2.69 diopters to −0.88 diopters, which suggests a potential enhancement in visual acuity and clarity (Figure 6).

#### 3.4.5. Topographic Astigmatism Changes

**Figure 7:**
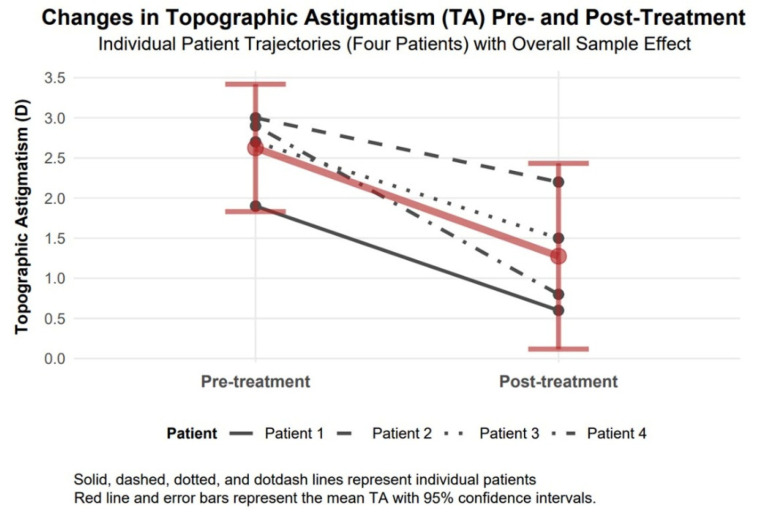
Changes in topographic astigmatism 6 months after topography-guided custom ablation treatment compared to pre-treatment (N = 4).

Topographic astigmatism demonstrated a significant improvement of 1.35 diopters in absolute value (95% confidence interval: 0.48 to 2.22, Cohen’s d = 2.16), with a large effect size, reducing from 2.63 diopters to 1.28 diopters, implying improved corneal regularity (Figure 7).

#### 3.4.6. Mean Keratometry Changes

**Figure 8:**
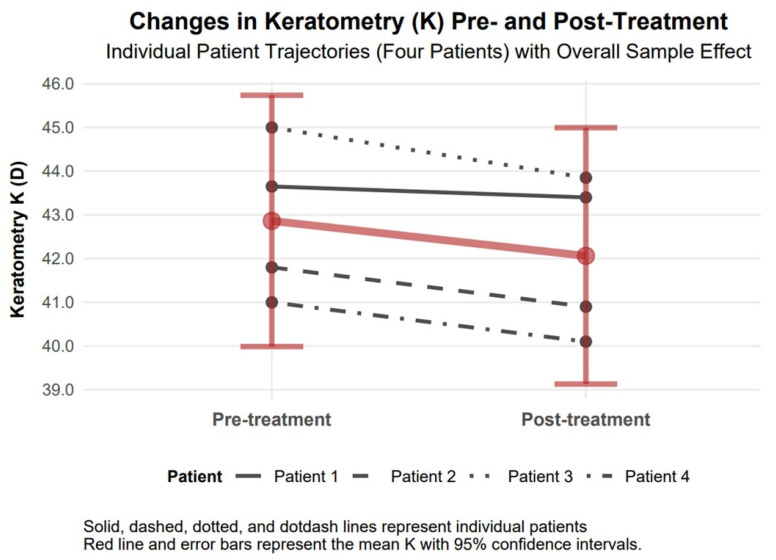
Changes in mean keratometry (K) 6 months after topography-guided custom ablation treatment compared to pre-treatment (N = 4).

The mean keratometry (K) exhibited a statistically significant reduction in absolute value by 0.80 diopters (95% confidence interval: 0.19 to 1.41), decreasing from 42.86 diopters to 42.06 diopters six months following treatment, indicative of notable corneal flattening. A Cohen’s d value of 0.44 revealed a moderate effect (Figure 8).

#### 3.4.7. Corneal Thickness Changes

**Figure 9:**
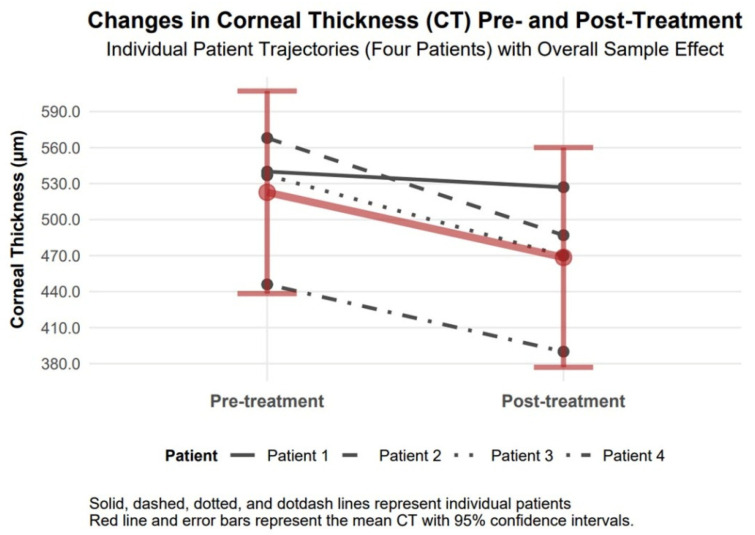
Changes in corneal thickness 6 months after topography-guided custom ablation treatment compared to pre-treatment (N = 4).

Corneal thickness decreased by −54.20 µm (95% CI −101.00 to −7.56, Cohen’s d = −0.98), declining from 522.75 µm to 468.55 µm (Figure 9).

#### 3.4.8. RMS Higher-Order Aberration Changes

**Figure 10:**
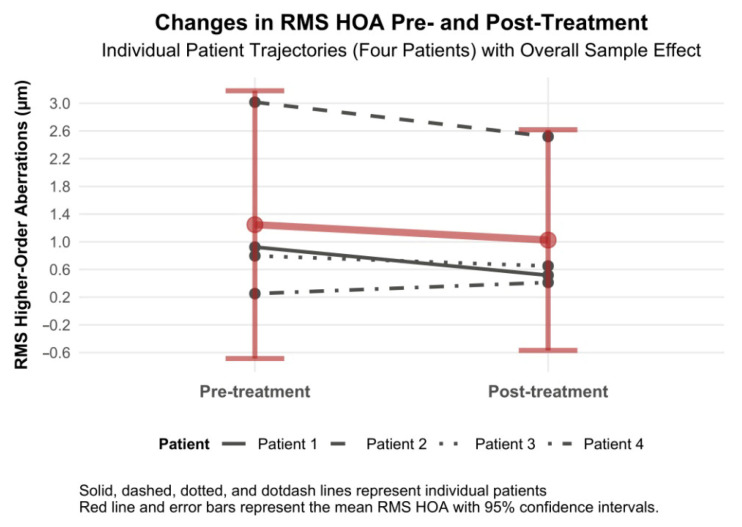
Changes in RMS higher-order aberrations 6 months after topography-guided custom ablation treatment compared to pre-treatment (N = 4).

RMS higher-order aberrations decreased by −0.22 µm (95% CI −0.69 to 0.25, Cohen’s d = −0.20), which had a positive effect on the quality of vision (Figure 10). We wish to highlight patients with RMS HOAs who experienced RMS HOAs reduction as a result of laser treatment (patients 1, 2 and 3). In one patient (patient 4) with a normal range of HOA at baseline, there was a slight increase, but still within the normal range (i.e., to 0.500 µm) (Figure 10).

**Figure 11:**
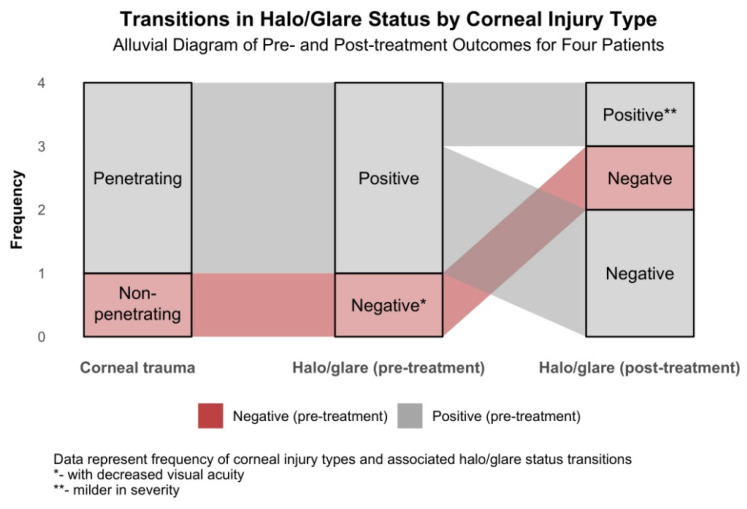
Changes in Halo/Glare Status 6 months after topography-guided custom ablation treatment compared to pre-treatment (N = 4) by corneal injury type.

Figure 11 illustrates the transitions in Halo/Glare Status for four patients with post-traumatic corneal irregularities at 6 months following topography-guided custom ablation treatment. Baseline characteristics presented in Table 1 reveal that three patients (Patients 1–3) presented with penetrating corneal wounds (75.0%), while one patient (Patient 4) had non-penetrating corneal trauma (25.0%). Before treatment, the three patients with penetrating wounds exhibited positive Halo/Glare Status. In contrast, the patient with non-penetrating corneal trauma demonstrated a negative Halo/Glare Status associated with decreased visual acuity. At the 6-month follow-up, two of the three patients with penetrating wounds changed from positive to negative Halo/Glare Status, and the third improved to a milder severity status. The patient with non-penetrating corneal trauma maintained a negative Halo/Glare Status, with noted improvements in both UDVA and BCVA. These findings suggest that topography-guided custom ablation treatment may effectively alleviate Halo/Glare symptoms in patients with diverse profiles of traumatic corneal irregularities.

**Figure 12:**
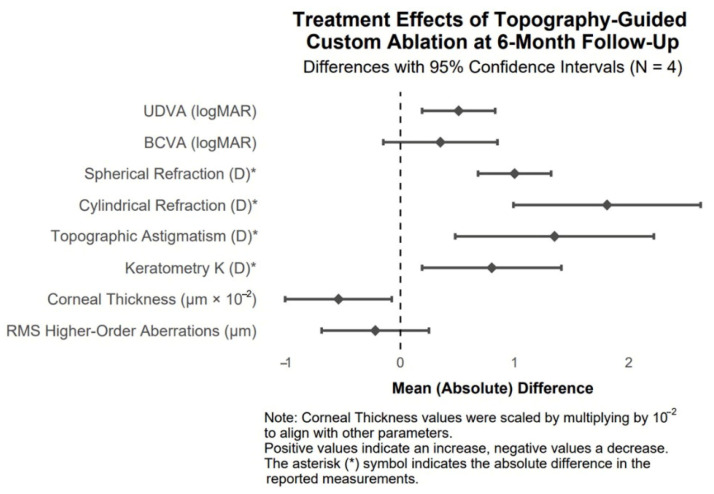
Forest plot of mean treatment effects of topography-guided custom ablation on clinical parameters at 6 months post-treatment in patients with post-traumatic corneal irregularities (N = 4).

The effects of topography-guided custom ablation on clinical parameters at 6 months post-treatment in patients with post-traumatic corneal irregularities are visualized as mean differences with 95% confidence intervals in the forest plot (Figure 12).

## 4. Discussion

The cornea is responsible for two-thirds of the refractive power of the eye. Adequate corneal translucency, along with optimum curvature and symmetry, enables light to enter the eye and form a real image on the retina [13]. Although surface regularization is derived from elevation maps, clinical refraction and wavefront analysis data determine the target corneal surface. TG ablation delivers an excimer laser beam to flatten steep areas of the cornea and steepen the flat surfaces. This combination of myopic and hyperopic ablation minimizes corneal stroma loss while maintaining a typical prolate shape (steeper centrally and flatter toward the periphery). TG ablation centration is based on the cornea apex rather than the pupil centre, eliminating the kappa angle issue.

It should be noted, though, that TCAT only corrects corneal aberrations; lenticular HOAs remain, which may degrade visual quality [2,14]. Therefore, clinical refraction is vital in laser vision correction. Occasionally, the magnitude and axis of astigmatism in refractive data may differ from the topographic data. Hence, TG treatments are based on corneal topography and refractive eye measurements, as topography alone does not provide data on spherical error [2,15]. The corneal surface regularization procedures our patients underwent utilized a combination of clinical refraction and topography data, specifically topography-modified refraction (TMR).

Additionally, the Schwind Amaris software supports TG treatment after accounting for anterior and posterior corneal astigmatism and topographic abnormalities that create HOAs [16]. Several studies comparing WFO and TG laser treatments have demonstrated similar efficacy in improving UDVA and astigmatism. However, Kim et al. showed that while both WFO and TG transepithelial photorefractive keratectomy effectively corrected myopia and myopic astigmatism, there was less induction of total corneal HOAs and spherical aberration after TG ablation [17]. Zhang et al. obtained a similar result after comparing three ablation methods [small incision lenticule extraction (SMILE), wavefront-optimized FS-LASIK, TCAT FS-LASIK] for myopia and myopic astigmatism. All interventions yielded comparable visual outcomes, but the corneal visual quality was best after TCAT FS-LASIK [18].

Excimer laser guided by the individual’s corneal topography map reshapes the corneal stromal layer. This surgical technique targets each portion of corneal tissue, correcting corneal shape irregularities and optimizing corneal curvature. Qualifying a patient for laser treatment typically involves vision tests, which can be performed using a pinhole or a rigid gas-permeable contact lens (RGP), such as a scleral lens.

Post-traumatic corneal scarring changes corneal topography and contributes to vision defects that are difficult to correct with standard methods. Many such patients are referred for penetrating keratoplasty. However, even deep scars that reach the corneal centre can respond to laser treatment, which regularizes the corneal surface, reduces vision defects, and improves visual quality. The corneal thinning observed in our study does not cause its destabilization, as it is within the range typical for surface laser treatments. Furthermore, the scarring process causes the cornea to stiffen rather than weaken [19]. To date, only a few reports have indicated that treatment with TCAT may enhance visual acuity [20,21]. The results obtained in our patients confirm these observations, as they achieved significant improvement in UDVA and BCVA. Avoiding invasive and high-risk procedures is extremely important. The first-stage laser treatment primarily aims to regularize the corneal surface but is not intended to correct the refractive defect, although some undercorrection is typically achieved. Second-stage laser treatment (solely refractive action of the laser) may be considered for residual defects, whose correction improves visual acuity. Our patients had all been informed that two-stage laser treatment might prove necessary. However, the first approach produced satisfactory results.

The available data support observations of comparable therapeutic responses to TCAT across patients with penetrating (Patients 1–3) and non-penetrating (Patient 4) corneal injuries. Visual assessment of study parameters, such as improvement in visual acuity and reduction in refractive and topographic astigmatism, as presented in Figure 3, Figure 4, Figure 5, Figure 6, Figure 7, Figure 8, Figure 9 and Figure 10, demonstrates a similar trend slope in these parameters across injury types. However, the study’s sample size, with only one patient in the non-penetrating injury subgroup, precludes statistical comparisons, as subgroup analysis was beyond the study’s objectives.

The study’s small sample size of four patients constrains the statistical robustness, potentially leading to wider confidence intervals and limited generalizability. A larger sample would enhance statistical power. We believe the focused nature of this case series offers valuable preliminary evidence for clinicians managing similar patient profiles, particularly given the detailed patient-level data and pooled statistical trends presented. The six-month follow-up period is enough to display long-term outcomes, such as the durability of TCAT’s effects on corneal regularization and visual acuity. The inclusion of male patients only and the narrow age range limits our ability to generalize the results to other demographic groups, such as female patients or individuals outside this age group. Future studies with larger and more diverse samples, including female patients and a broader age range, are needed to validate these preliminary findings and further explore TCAT’s efficacy in heterogeneous populations.

## 5. Conclusions

According to our findings, topography-guided custom ablation treatment demonstrates highly significant efficacy in improving UDVA, cylindrical refraction, and topographic astigmatism in patients with post-traumatic corneal irregularities. Substantial changes and large effect sizes indicate potential benefits for visual function.

In numerical values, the mean UDVA, as measured by the Snellen chart, improved from 0.44 to 0.95, and the mean BCVA from 0.83 to 1.18. In absolute values, the mean spherical refraction improved by 1 diopter, cylindrical refraction by 1.81 diopters, and topographic astigmatism was reduced by 1.35 diopters. The mean keratometry, expressed in absolute values, was reduced by 0.8 diopters, a statistically significant change. The mean RMS HOA was reduced by 0.22 µm, resulting in a significant improvement in the visual quality of treated patients.

TG ablation following surgical debridement and corneal wound healing can be a valuable method for treating corneal trauma-induced astigmatism. Current lasers, such as the Schwind laser, allow the treatment of irregular corneal astigmatism based on customized ablation and consider HOAs and corneal HOAs. TCAT reduces irregular corneal astigmatism, regularizes the corneal surface, corrects the defect, and improves visual acuity and quality through laser correction of HOAs, especially coma. The enhanced visual acuity and reshaped corneal surface enable the selection of spectacle correction and, in many cases, also eliminate the need for invasive procedures such as corneal transplantation.

## Figures and Tables

**Table 1:** Baseline characteristics of patients with post-traumatic corneal irregularities (N = 4).

Characteristic	N	Value
**Demographics**		
Male	4	−100.00%
Age (years)		
Mean (SD)		44.00 (3.56)
Range [Min, Max]		[41.00, 48.00]
**Injury Characteristics**		
Injury Characteristics	4	
Non-penetrating Corneal Trauma		1 (25.0%)
Penetrating Wound of the Cornea		3 (75.0%)
**Treatment History**		
Emergency treatment:	4	
Surgical Treatment—Suturing of the Corneal Wound		3 (75.0%)
No Surgical Treatment		1 (25.0%)
Laser Treatment:	4	
Corneal Surface Regularization by TCAT Method		4 (100.0%)
**Clinical Parameters**		
UDVA (decimal notation)	4	
Mean (SD)		0.44 (0.26)
Range [Min, Max]		[0.15, 0.70]
BCVA (decimal notation)	4	
Mean (SD)		0.83 (0.24)
Range [Min, Max]		[0.50, 1.00]
Spherical Refraction (D)	4	
Mean (SD)		0.88 (1.64)
Range [Min, Max]		[−1.50, 2.25]
Cylindrical Refraction (D)	4	
Mean (SD)		−2.69 (0.72)
Range [Min, Max]		[−3.75, −2.25]
Topographic Astigmatism (D):	4	
Mean (SD)		2.63 (0.50)
Range [Min, Max]		[1.90, 3.00]
Keratometry (D):	4	
Mean (SD)		42.86 (1.81)
Range [Min, Max]		[41.00, 45.00]
Corneal Thickness (µm):	4	
Mean (SD)		522.75 (53.04)
Range [Min, Max]		[446.00, 568.00]
RMS Higher-Order Aberrations (µm):	4	
Mean (SD)		1.25 (1.22)
Range [Min, Max]		[0.25, 3.02]
Halo/Glare Status:	4	
Positive		3 (75.0%)
Negative, with Decreased Visual Acuity		1 (25.0%)

Note: N—sample size, Bold in the table—variable groups headings

**Table 2:** Pre-TCAT results, refractive maps, and Zernike analyses.

Variable	Patient 1	Patient 2	Patient 3	Patient 4
UDVA	0.7	0.15	0.3	0.6
BCVA	1.0	0.5	0.8	1.0
Autorefractometry	+2.25/−2.25 ax 47	−1.50/−2.25 ax 151	+1.50/−3.75 ax 6	+1.25/−2.50 ax 174
Topographic astigmatism	1.9 D ax 25.5	3.0 D ax 151	2.7 D ax 11.2	2.9 D ax 175
Keratometry	K1 42.7 K2 44.6	K1 40.3 K2 43.3	K1 43.6 K2 46.4	K1 39.5 K2 42.5
Mean Keratometry	43.65	41.8	45.0	41.0
Pachymetry	540 µm	568 µm	537 µm	446 µm
RMS HOA	0.923 µm	3.016 µm	0.797 µm	0.251 µm
Halo/glare	(+)	(+)	(+)	No, but visual acuity decreased

Note: ax—cylinder axis

**Table 3:** Post-TCAT results, refractive maps, and Zernike analyses.

Variable	Patient 1	Patient 2	Patient 3	Patient 4
UDVA	1.0	0.9	0.7	1.2
BCVA	1.0	1.2	1.0	1.5
Autorefractometry	+1.50/−1.00 ax 69	−0.50/−0.75 ax 138	−0.25/−1.50 ax 107	+0.25/−0.25 ax 178
Topographic astigmatism	0.6 D ax 135	2.2 D ax 114	1.5 D ax 119	0.8 D ax 10
Keratometry	K1 43.1 K2 43.7	K1 39.8 K2 42.0	K1 43.1 K2 44.6	K1 39.7 K2 40.5
Mean Keratometry	43.4	40.9	43.85	40.1
Pachymetry	527 µm	487 µm	470 µm	390 µm
RMS HOA	0.516 µm	2.519 µm	0.650 µm	0.412 µm
Halo/glare	(-)	Milder	(-)	(-)

**Table 4:** Changes in numerical clinical parameters 6 months after topography-guided custom ablation treatment compared to pre-treatment (N = 4).

Parameter	Mean (Absolute) Difference	SD of Difference	SE of Difference	95% CI Lower	95% CI Upper	SD Pre-Treatment	SD Post-Treatment	SD Pooled	Cohen’s d
UDVA (decimal notation)	0.51	0.2	0.1	0.19	0.83	0.26	0.21	0.23	2.2
BCVA (decimal notation)	0.35	0.31	0.16	−0.15	0.85	0.24	0.24	0.24	1.48
Spherical Refraction (D) *	1	0.2	0.1	0.68	1.32	0.43	0.6	0.52	1.92
Cylindrical Refraction (D) *	1.81	0.52	0.26	0.99	2.63	0.72	0.52	0.63	2.89
Topographic Astigmatism (D) *	1.35	0.55	0.27	0.48	2.22	0.5	0.73	0.62	2.16
Keratometry K (D) *	0.8	0.39	0.19	0.19	1.41	1.81	1.84	1.82	0.44
Corneal Thickness (µm)	−54.2	29.3	14.7	−101	−7.56	53	57.5	55.3	−0.98
RMS Higher-Order Aberrations (µm)	−0.22	0.3	0.15	−0.69	0.25	1.22	1	1.11	−0.2

Notes: the mean difference quantifies the change in a parameter from pre-treatment to the 6-month follow-up. It is calculated as the difference between the follow-up value and the pre-treatment value; positive values indicate an increase, and negative values indicate a decrease. Cohen’s d effect size interpretation: small (0.2), medium (0.5), large (0.8). Confidence intervals may be wide due to the small sample size (N = 4). The asterisk (*) indicates the absolute difference in the reported measurements.

## Data Availability

The original contributions presented in this study are included in the article.

## Supplementary Materials

The following supporting information can be downloaded at: https://www.mdpi.com/article/10.3390/biomedicines13081818/s1, Figure S1: Patient 1—the top of the figure shows the scans of the Pentacam test: top-left—before treatment; top-middle—after treatment; top-right—the difference in refractive results before and after treatment. The bottom part shows the corneal tomography image. The white arrow indicates the site of injury. Figure S2: Patient 2—the top of the figure shows the scans of the Pentacam test: top-left—before treatment; top-middle—after treatment; top-right—the difference in refractive results before and after treatment. The bottom part shows the corneal tomography image. The white arrow indicates the site of injury. Figure S3: Patient 4—the top of the figure shows the scans of the Pentacam test: top-left—before treatment; top-middle—after treatment; top-right—the difference in refractive results before and after treatment. The bottom part shows the corneal tomography image. The white arrow indicates the site of injury.

## References

[1] Zagórski Z., Kuhn F. (2014). Zaopatrzenie rany rogówki w ramach ostrego dyżuru okulistycznego. Okul. Po Dyplomie.

[2] Ramamurthy S., Soundarya B., Sachdev G.S. (2020). Topography-guided treatment in regular and irregular corneas. Indian J. Ophthalmol.

[3] Idris L., Khandekar R., Ahad M. (2020). Topography guided custom ablation treatment for residual refractive error after keratoplasty. J. EuCornea.

[4] Benchimol E.I., Smeeth L., Guttmann A., Harron K., Moher D., Petersen I., Sorensen H.T., von Elm E., Langan S.M., Committee R.W. (2015). The REporting of studies Conducted using Observational Routinely-collected health Data (RECORD) statement. PLoS Med..

[5] Gagnier J.J., Kienle G., Altman D.G., Moher D., Sox H., Riley D. (2013). The CARE Guidelines: Consensus-based Clinical Case Reporting Guideline Development. Glob. Adv. Health Med..

[6] R Core Team (2024). R: A Language and Environment for Statistical Computing.

[7] Brunson J.C., Read Q.D. (2023). “ggalluvial: Alluvial Plots in ‘ggplot2’.” R Package Version 0.12.5. https://corybrunson.github.io/ggalluvial/.

[8] Makowski D., Lüdecke D., Patil I., Thériault R., Ben-Shachar M., Wiernik B. (2023). Automated Results Reporting as a Practical Tool to Improve Reproducibility and Methodological Best Practices Adoption. CRAN. https://easystats.github.io/report/.

[9] Sjoberg D., Whiting K., Curry M., Lavery J., Larmarange J. (2021). Reproducible Summary Tables with the gtsummary Package. R J..

[10] Wickham H. (2016). ggplot2: Elegant Graphics for Data Analysis.

[11] Wickham H., François R., Henry L., Müller K., Vaughan D. (2023). dplyr: A Grammar of Data Manipulation. R Package Version 1.1.4. https://CRAN.R-project.org/package=dplyr.

[12] Wickham H., Vaughan D., Girlich M. (2024). tidyr: Tidy Messy Data. R Package Version 1.3.1. https://CRAN.R-project.org/package=tidyr.

[13] Akincioglu D., Ozge G., Gokce G., Ayyildiz O., Karaca U., Mutlu F.M. (2021). Scheimpflug imaging of the anterior segment following simultaneous cross-linking with topography-guided custom ablation treatment for keratoconus. Arq. Bras. De Oftalmol..

[14] Jain A.K., Malhotra C., Pasari A., Kumar P., Moshirfar M. (2016). Outcomes of topography-guided versus wavefront-optimized laser in situ keratomileusis for myopia in virgin eyes. J. Cataract. Refract. Surg.

[15] Kanellopoulos A.J. (2016). Topography-modified refraction (TMR): Adjustment of treated cylinder amount and axis to the topography versus standard clinical refraction in myopic topography-guided LASIK. Clin. Ophthalmol..

[16] Lobanoff M., Stonecipher K., Tooma T., Wexler S., Potvin R. (2020). Clinical outcomes after topography-guided LASIK: Comparing results based on a new topography analysis algorithm with those based on manifest refraction. J. Cataract. Refract. Surg..

[17] Kim S., Na S., Choi S., Choi S.H. (2024). Comparison of Outcomes after Wavefront-optimized and Topography-guided Transepithelial Photorefractive Keratectomy. Korean J. Ophthalmol..

[18] Zhang Y., Sun X., Chen Y. (2022). Comparison of Corneal Optical Quality After SMILE, Wavefront-Optimized LASIK and Topography-Guided LASIK for Myopia and Myopic Astigmatism. Front. Med..

[19] Raghunathan V.K., Thomasy S.M., Strom P., Yanez-Soto B., Garland S.P., Sermeno J., Reilly C.M., Murphy C.J. (2017). Tissue and cellular biomechanics during corneal wound injury and repair. Acta Biomater..

[20] Kanellopoulos A.J. (2012). The management of cornea blindness from severe corneal scarring, with the Athens Protocol (transepithelial topography-guided PRK therapeutic remodeling, combined with same-day, collagen cross-linking). Clin. Ophthalmol..

[21] Arfaj K.A., Jain V., Hantera M., El-Deeb M.W., Rushod A.A., Nair A.G., Pineda R. (2011). Phototherapeutic keratectomy outcomes in superficial corneal opacities. Ophthalmol. Eye Dis..

