# Supplementary material for: Topography-Guided Custom Ablation Treatment for Post-Traumatic Corneal Irregularities—Case Reports
Source: Biomedicines. 2025 Jul 24;13(8):1818. doi: 10.3390/biomedicines13081818 (PMC12383987; doi:10.3390/biomedicines13081818)
Supplement: Supplementary file 1 [file biomedicines-13-01818-s001.zip › biomedicines-3707714-supplementary.pdf]

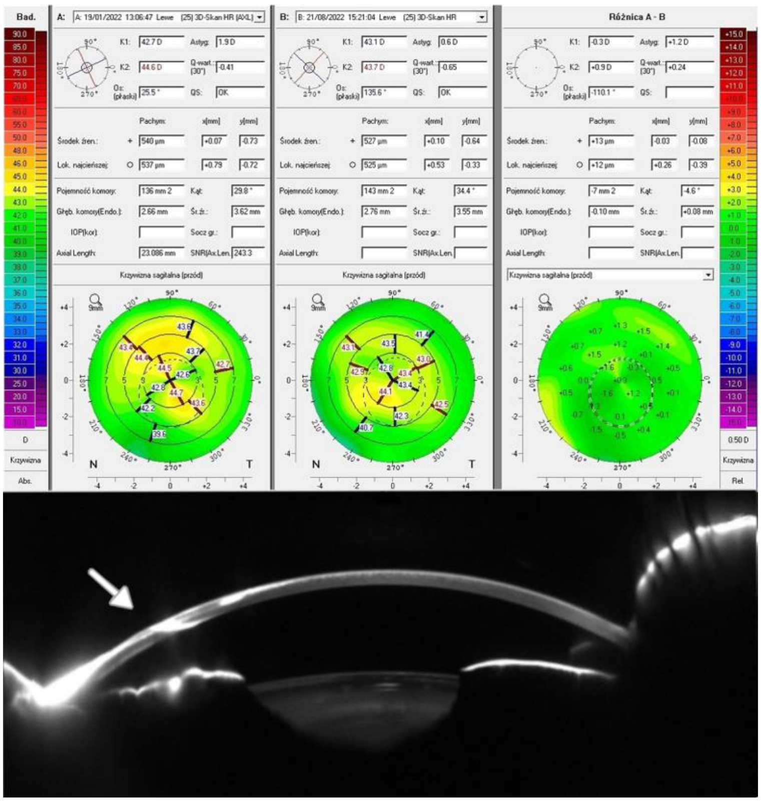

Figure S1: Patient 1—the top of the figure shows the scans of the Pentacam test: top-left—before treatment; top-middle—after treatment; top-right—the difference in refractive results before and after treatment. The bottom part shows the corneal tomography image. The white arrow indicates the site of injury.

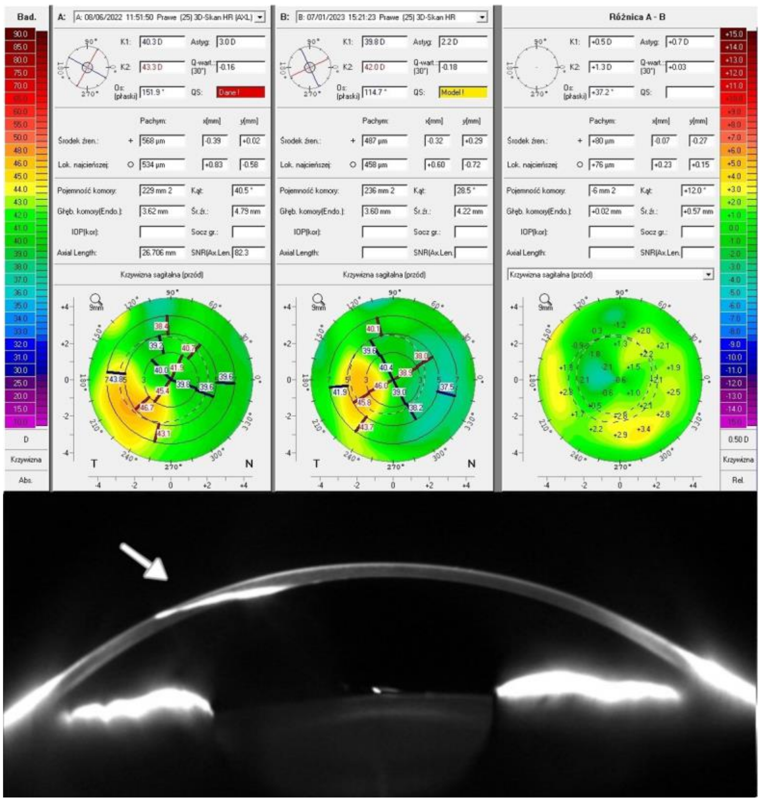

Figure S2: Patient 2—the top of the figure shows the scans of the Pentacam test: top-left—before treatment; top-middle—after treatment; top-right—the difference in refractive results before and

after treatment. The bottom part shows the corneal tomography image. The white arrow indicates the site of injury.

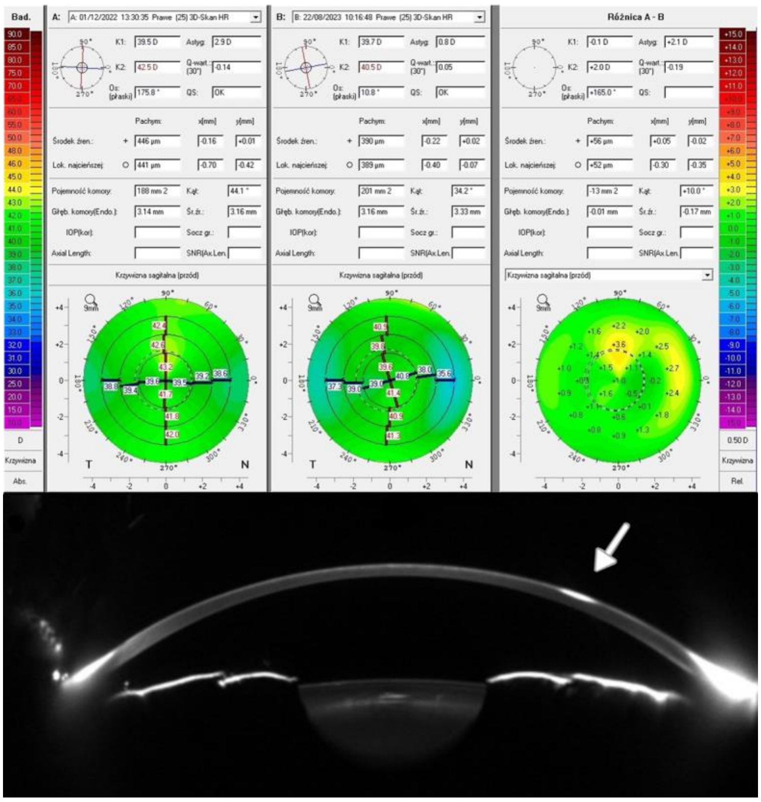

Figure S3: Patient 4—the top of the figure shows the scans of the Pentacam test: top-left—before treatment; top-middle—after treatment; top-right—the difference in refractive results before and after treatment. The bottom part shows the corneal to-mography image. The white arrow indicates the site of injury.
